# Pilates and Kinesiotaping for Neck Pain, Disability and Cervical Function in Office Workers: A Randomised Controlled Trial

**DOI:** 10.1155/prm/1954204

**Published:** 2026-07-23

**Authors:** Abdulkadir Göz, Ali Mutlu, Evrim Göz

**Affiliations:** ^1^ Department of Physical Therapy and Rehabilitation, Tarsus State Hospital, Mersin, Turkey; ^2^ Department of Physiotherapy and Rehabilitation, Graduate School of Health Sciences, Hacettepe University, Ankara, Turkey, hacettepe.edu.tr; ^3^ Department of Physiotherapy and Rehabilitation, Faculty of Health Sciences, Tarsus University, Mersin, Turkey, tarsus.edu.tr

**Keywords:** chronic neck pain, kinesiotaping, office workers, Pilates

## Abstract

**Background:**

Prolonged computer use among office workers leads to postural disorders and musculoskeletal pain, particularly in the neck region. Pilates exercises and kinesiotaping are widely used interventions for the management of chronic neck pain.

**Aims:**

This study aimed to investigate the effects of Pilates exercises and kinesiotaping on neck pain, disability, cervical range of motion (CROM), neck muscle strength and core endurance in office workers with chronic neck pain.

**Methods:**

A randomised controlled trial was conducted with 30 office workers who completed the intervention. Participants were randomly assigned to either a Pilates group (*n* = 16) or a kinesiotaping group (*n* = 14). Both groups participated in an 8‐week, supervised Pilates programme, while the kinesiotaping group additionally received kinesiotaping applications to the cervical region. Assessments of neck pain, disability, CROM, muscle strength and core endurance were conducted pre‐ and postintervention.

**Results:**

Both groups demonstrated significant improvements in pain, disability, CROM, neck muscle strength and core endurance (*p* < 0.05). Between‐group comparisons revealed no significant differences in pain and disability outcomes (*p* > 0.05). However, an additional improvement in left cervical rotation CROM was observed in the kinesiotaping group (*p* = 0.001).

**Conclusions:**

Eight weeks of Pilates training significantly improved pain, disability and functional outcomes in office workers with chronic neck pain. Kinesiotaping provided limited additional benefits, primarily in rotational movements. From an occupational rehabilitation perspective, Pilates‐based exercises may support work‐related functional capacity and sustained work participation in sedentary occupations, while kinesiotaping may serve as a complementary modality. Further studies with larger samples are warranted to confirm these results.

**Trial Registration:** ClinicalTrials.gov identifier: NCT06262997

## 1. Introduction

Computer and internet technologies have become an indispensable part of daily and professional life. However, this has led to the adoption of a more sedentary lifestyle [1]. Increased computer use, particularly in offices, means employees spend long periods in front of screens, which leads to an increase in postural disorders and musculoskeletal problems [2]. Maintaining a static posture for extended periods can result in postural issues, including an increased anterior tilt of the head and increased kyphosis in the thoracic and lumbar regions. These issues primarily manifest as pain in the neck and shoulder areas [3].

Neck pain is a widespread health issue and one of the leading causes of work‐related absence and disability worldwide. It affects up to 40% of office workers [4]. The neck muscles play an important role in the mobility and stability of a healthy cervical spine. Studies show that muscle weakness and fatigue can lead to pain [5]. It is well established that 80% of spinal stability depends on muscle activity [6]. Decreased deep flexor muscle function and impaired activation of the upper trapezius, cervical extensors, anterior scalene muscles and sternocleidomastoid muscles have been reported to be associated with neck pain, causing various neuromuscular dysfunctions [7]. Consequently, individuals with neck pain frequently exhibit reduced cervical range of motion (CROM), muscle strength and endurance [5].

Exercise is an effective method of alleviating neck pain and improving functional limitations. It is therefore considered a fundamental component of rehabilitation programmes. The literature reports that endurance‐based exercises, scapular muscle re‐education and exercises that strengthen and stretch the muscles in the cervical and scapulothoracic regions are commonly used to treat chronic neck pain [8]. In recent years, Pilates exercises have become a popular choice for rehabilitating neck pain because they combine strengthening, stretching and proprioceptive exercises [9].

Pilates is a mind–body exercise method that increases muscle strength and flexibility, improves posture and develops core stability by controlling your breathing [10, 11]. Although its effectiveness has been extensively investigated in individuals with low back pain, evidence regarding its role in the management of chronic neck pain remains comparatively limited [12]. Recent systematic evidence suggests that Pilates may be a promising strategy for reducing pain and disability, although its superiority over other active exercise modalities is still being debated [13]. In addition to its potential pain relief effects, Pilates has been associated with favourable adaptations in cervical muscle morphology. For example, ultrasonographic assessments have demonstrated increased thickness of the semispinalis capitis muscle following Pilates training, suggesting enhanced structural support and stabilisation of the cervical region [9]. Furthermore, the method’s focus on controlled movement and breathing may offer broader clinical benefits, such as improving sleep quality and encouraging patients to adopt more active coping strategies in managing their chronic symptoms [14].

In recent years, kinesiotaping has become an increasingly popular method of managing neck pain. Kinesiotaping involves the application of elastic therapeutic tape to the skin using specific techniques designed to provide support to muscles and joints without substantially restricting range of motion. It has been proposed that kinesiotaping may facilitate soft tissue function, improve neuromuscular control and contribute to pain reduction [15]. Mechanistically, the effectiveness of kinesiotaping is attributed to its ability to lift the skin, which increases the interstitial space, improves blood and lymph circulation and alleviates pressure on sensory receptors [16]. This application provides sensory feedback that facilitates pain relief through the gate control theory and enhances proprioceptive awareness [17]. Kinesiotaping has also been shown to be an effective complementary treatment for musculoskeletal problems, neurological and rheumatological diseases, lymphoedema and various painful conditions [18, 19]. Current evidence indicates that kinesiotaping may contribute to reductions in pain intensity and improvements in functional outcomes among individuals with neck pain [2, 20]. It has been shown to be particularly effective in the neck and upper extremities during the first 5 days of application [16]. Furthermore, kinesiotaping helps to correct muscle alignment and stimulate contraction in weakened muscles, which could benefit various occupational groups who experience neck pain [17, 21].

Although both Pilates exercise and kinesiotaping have shown beneficial effects in individuals with chronic neck pain, evidence comparing their effectiveness remains limited. In addition, previous studies have mainly focused on pain and disability, while outcomes such as CROM, neck muscle strength and core endurance have been less frequently investigated. Office workers represent a high‐risk population due to prolonged sitting and sustained postural demands. Therefore, this randomised controlled trial aimed to compare the effects of Pilates exercise alone and Pilates combined with kinesiotaping on neck pain, disability, CROM, neck muscle strength and core endurance in office workers with chronic neck pain.

## 2. Method

This study employed a randomised controlled design, with both the assessor and statistician blinded to group allocation. The study was conducted and reported in accordance with the CONSORT statement. This design was chosen for its ability to assess the efficacy of interventions while minimising the influence of potential confounding variables. The first researcher conducted the Pilates and kinesiotaping interventions, the second researcher performed the pre‐ and postintervention assessments, and the third researcher carried out the statistical analyses. The second and third researchers remained blinded to group allocation throughout the study. The study was conducted between February and August 2024 with office workers employed at Tarsus University.

### 2.1. Participants and Assessments

**FIGURE 1:**
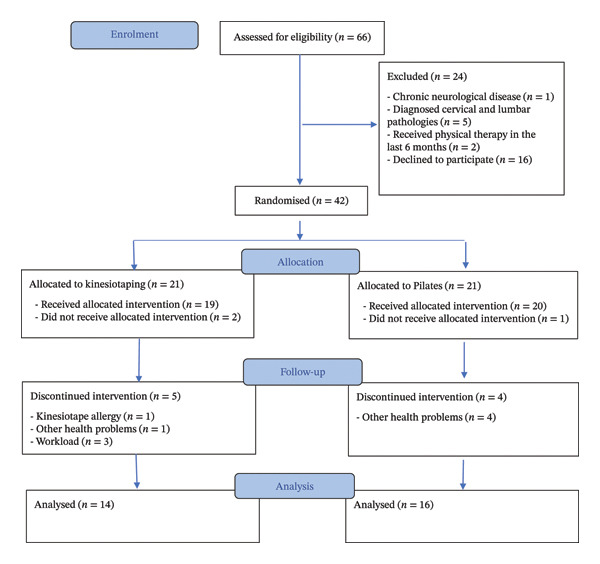
Flowchart of the study.

Participants were screened during the initial interview for eligibility according to the inclusion and exclusion criteria. A total of 42 eligible participants who provided written informed consent were randomly assigned to either the Pilates or kinesiotaping group using a computer‐generated permuted block randomisation sequence with a block size of four generated by the Study Randomizer software (https://www.studyrandomizer.com). The randomisation procedure was performed by the researcher responsible for administering the interventions. Following randomisation, baseline assessments were conducted by a researcher blinded to group allocation. Pain intensity was assessed using the Visual Analogue Scale (VAS); neck disability, using the Neck Disability Index (NDI); muscle strength, using a push–pull dynamometer; CROM, using a digital inclinometer; and core muscle strength and endurance, using sit‐up and prone bridge tests. Initially, 21 participants were allocated to each group; however, due to dropouts during the intervention period, the study was completed by 16 participants in the Pilates group and 14 participants in the kinesiotaping group (Figure 1). All participants received 8 weeks of Pilates training.

### 2.2. Intervention Design

#### 2.2.1. Pilates Group

Participants received one‐hour Pilates training sessions twice a week for 8 weeks. These sessions were conducted in groups of three to four people under the supervision of a physiotherapist. The introductory session covered basic Pilates principles. The physiotherapist demonstrated movements and provided guidance using tactile, verbal and visual cues [22]. From the fourth week onwards, the level of exercise, body positions and use of exercise balls and bands were gradually increased according to participants′ performance (Supporting Figures 1–6). Training consisted of a warm‐up phase, 40 min of basic exercises and a cool‐down phase (Table 1). The programme was conducted by a physiotherapist with 13 years’ experience and Pilates certification.

**TABLE 1:** Pilates training.

**Programme 1–4 weeks**		

Warm‐up exercises (10 min)	Breathing Centring Neck, trunk and extremity mobility exercises Roll down, lateral roll down	

Pilates exercises (40 min)	‘Shoulder drop′ ‘Chestlift 1‐2’ ‘Hundred’ ‘Single Leg Circles’ ‘Single Leg Stretch’ ‘Side to side’ ‘Shoulder Bridge 1’ ‘Book Opening’ and Stretch	‘Up/Down Side Kicks’ ‘Side Lift 1‐2’ ‘Spine Stretch Forward’ ‘Spine Twist’ ‘Press Up’ ‘Single Leg Kicks’ ‘Scarecrow 1‐2‐3’ ‘Modified swimming’

Cool‐down exercises (10 min)	‘Child’s Pose’ ‘Cat Stretch’ ‘Mermaid 1’ ‘Dumb waiter’ ‘Cleopatra’ ‘Lunges’ ‘Standing Balance’	

Total 60 min		

**Programme 5–8 weeks**		

Warm‐up exercises (10 min)	Breathing Centring Neck, trunk and extremity mobility exercises Roll down, lateral roll down	

Pilates exercises (40 min)	‘Shoulder drop’ ‘Chestlift 1‐2’ ‘Hundred’ ‘Single Leg Circles with band’ ‘Crisscross’ ‘Side to side’ ‘Shoulder Bridge 2’ ‘Book Opening’ and Stretch	‘Clam’ ‘Side Lift 1‐2’ ‘Spine Stretch Forward’ ‘Spine Twist’ ‘Press Up’ ‘Single Leg Kicks’ ‘Scarecrow 1‐2‐3’ ‘Modified swimming quadruped’

Cool‐down exercises (10 min)	‘Child’s Pose’ ‘Cat Stretch’ ‘Mermaid 2’ ‘Dumb waiter with band’ ‘Cleopatra with band’ ‘Lunges with balls’ ‘Standing Balance’	

Total 60 min		

#### 2.2.2. Kinesiotaping Group

**FIGURE 2:**
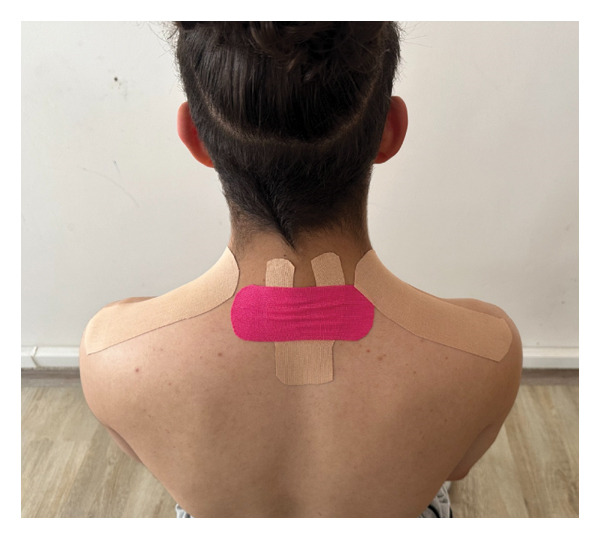
Kinesiotaping method.

Participants in the kinesiotaping group completed the identical supervised Pilates programme described for the Pilates group. Additionally, at the end of each session, Y and I tapes (Kinesio Tex Gold FP‐5 cm) were applied to the longissimus cervicalis muscle, and an I tape was applied to the upper trapezius (Figure 2). As the exercise programme was performed twice weekly for eight weeks, a total of 16 kinesiotaping applications were administered. The tape remained in place until the subsequent session and was renewed at each visit by the same physiotherapist.

### 2.3. Population and Sample

The study population consisted of office workers aged 18 and over with neck pain at Tarsus University. Sample size calculation using OpenEpi software, based on a study by Cazzotti et al. reporting a 6.59‐point reduction in disability scores after Pilates [23], indicated a total of 40 participants (≥ 20 per group) to achieve 95% confidence, 90% power and α = 0.05. Taking into account the possibility of participants being excluded from the study, a total of 42 people were planned to be included, comprising 21 kinesiotaping participants and 21 Pilates participants. A statistical power of 90% was selected to reduce the probability of Type II error and to increase confidence in detecting clinically meaningful differences between groups.

### 2.4. Inclusion/Exclusion Criteria

Participants aged between 18 and 65 years who had worked in an office for at least one year, used a computer at a desk for at least three hours per day and had experienced neck pain for more than six weeks (VAS > 3/10) were included in the study. Those with chronic neurological or rheumatological diseases, who had undergone physical therapy in the last 6 months; who had a history of spinal trauma or surgery; who had been diagnosed with cervical or lumbar pathologies; who had Pilates or kinesiotaping experience in the last 6 months; or who declined to participate in the study were excluded. Participants who withdrew from the study of their own accord after joining, whose general health status changed to a degree that would affect the study, or who exhibited an allergic reaction to kinesiotaping application were also excluded.

### 2.5. Measurement Tools

Primary outcomes of the study were neck pain intensity and neck‐related disability, assessed using the VAS and the NDI, respectively. Secondary outcomes included CROM, neck muscle strength and core muscle endurance.

#### 2.5.1. Collection of Demographic Data

Participants were asked about characteristics such as age, gender, occupation, educational status, height, weight, body mass index (BMI), how long they had experienced neck pain for, whether they were taking medication, and their habits with regard to smoking, alcohol consumption and exercise.

#### 2.5.2. VAS

A VAS scale consisting of a 10‐cm line was used to assess the intensity of neck pain. Participants were asked to mark a point on the line corresponding to their perceived pain intensity (0 = *no pain*, 10 = *most severe pain*), and this value was accepted as their pain intensity [24].

#### 2.5.3. NDI

The NDI is used to assess the impact of neck pain on daily living activities. This validated and reliability‐tested scale consists of ten sections: pain intensity; personal care; lifting; reading; headaches; concentration; work life; driving; sleep; and leisure activities. Each section is scored between 0 (*no disability*) and 5 (*complete disability*). Scores obtained from the scale range from 0 to 50, with an increase in score indicating an increase in level of disability [25, 26].

#### 2.5.4. Muscle Strength

Muscle strength in the neck region (neck flexion, extension and lateral flexion) was assessed using a baseline analogue hydraulic push–pull dynamometer. Participants were asked to perform the movement with maximum force, without using compensation mechanisms. The researcher then applied resistance in the opposite direction to ensure that no joint movement occurred and to achieve an isometric contraction [27]. The measurements were repeated twice, and the averages were taken.

#### 2.5.5. CROM

Movement of the neck joint (flexion, extension, lateral flexion and rotation) was measured using a Baseline Digital Inclinometer [28].

#### 2.5.6. Core Endurance Assessment

Core endurance was included as a secondary outcome because impaired trunk stability and reduced endurance of the lumbopelvic musculature have been associated with altered postural control and increased mechanical loading of the cervical region. Since Pilates primarily targets core stability and postural control, assessment of core endurance was considered relevant for evaluating the broader functional effects of the intervention.

##### 2.5.6.1. Prone Bridge Test

This test assesses the static endurance of the core muscles. For this test, individuals lie face down with their elbows flexed and lift their bodies upwards by placing their weight on their forearms and toes. The time taken to perform the test was recorded in seconds using a stopwatch, and the test was terminated when the test position was compromised. Each measurement was performed twice, and the average was recorded [29].

##### 2.5.6.2. Sit‐Ups Test

The dynamic endurance of the core muscles was assessed using the sit‐up test. Participants were asked to transition from a supine position with their knees flexed and their feet fixed to a body flexion position. The test duration was set at 30 s, and the number of repetitions completed within this time was recorded [29].

### 2.6. Ethical Approval and Consent to Participate

The study was approved by the Ethics Committee of Tarsus University on 25.05.2023 with decision number 2023/20. The Declaration of Helsinki was followed in the study. All participants signed informed consent.

### 2.7. Statistical Analysis

The obtained data were analysed using SPSS 22.0 for Windows (IBM Corporation) software. The normality of the variables was determined using Kolmogorov–Smirnov and Shapiro–Wilk tests, as well as histogram analysis. The results were presented descriptively using frequency values and percentage intervals, along with the median (interquartile range) values obtained from the measurements. For the analytical evaluation, the Mann–Whitney U test was performed to compare the two groups, as the parametric conditions were not met. The Wilcoxon test was used to examine changes within the groups [30]. Analyses were conducted on participants who completed both baseline and postintervention assessments (per‐protocol analysis). The significance value was set at *p* < 0.05.

## 3. Results

A total of 42 participants were initially enrolled and randomised. During the intervention period, 12 participants were lost to follow‐up or withdrew from the study for various reasons, including one participant in the kinesiotaping group who developed an allergic skin reaction related to the tape application and was withdrawn according to the predefined exclusion criteria. Consequently, the final analyses were performed on 30 participants who completed the study (Figure 1). Their clinical and demographic data are shown in Table 2. The groups were similar in terms of their demographic and clinical characteristics (*p* > 0.05).

**TABLE 2:** Comparison of demographic and clinical characteristics between groups.

	Kinesiotaping group *n* = 14	Pilates group *n* = 16	*p*
Age (year)	36.5 (33.0–43.0)	39.5 (34.75–44.75)	0.210
Height (m)	1.62 (1.61–1.71)	1.65 (1.61–1.68)	0.650
Weight (kg)	63.0 (59.0–74.0)	62.50 (60.0–74.0)	0.850
BMI (kg/m^2^)	23.85 (22.01–25.26)	23.80 (21.72–26.82)	0.770
VAS (score)	6.4 (4.7–7.4)	6.0 (4.8–0.1)	0.490
Duration of pain (month)	48.0 (21.0–60.0)	36.0 (36.0–60.0)	0.700
NDI (score)	12.5 (8.0–12.5)	13 (9.25–16.25)	0.480
Gender (F/M, *n*)	12/2	13/3	—
Exercise (yes/no, *n*)	1/13	2/14	—
Smoking (yes/no, *n*)	2/12	2/14	—

*Note:* Data are expressed as the median (interquartile range) and were compared using the Mann–Whitney U test. F, female; M, male.

Abbreviations: BMI, body mass index; NDI, Neck Disability Index; VAS, Visual Analogue Scale.

We performed the Wilcoxon test to assess changes over time and detected statistically significant improvements in pain and neck disability (*p* < 0.001), which was the primary outcome of the study. After the interventions, both groups showed improvements in terms of VAS and NDI values (*p* < 0.05, Table 3). However, no significant differences were observed between groups regarding improvements in pain or neck disability (*p* > 0.05, Table 3).

**TABLE 3:** Comparison of VAS and NDI parameters over time.

Outcome measure	Kinesiotaping group (*n* = 14) median (IQR)	Pilates group (*n* = 16) median (IQR)	*p* intergroup
VAS, score			
Baseline	6.4 (4.7–7.4)	6.0 (4.8−6.1)	
8 weeks	2.05 (1.23–4.0)	2.15 (1.70–3.90)	
Δ	−3.1	−3.0	0.493
*p* intragroup	0.002^∗^	0.001^∗^	
NDI, score			
Baseline	12.5 (8.0–12.5)	13 (9.25–16.25)	
8 weeks	7.5 (4.75–12.25)	8 (5.0–10.75)	
Δ	−5.5	−5.0	0.478
*p* intragroup	0.002^∗^	0.015^∗^	

*Note:* *p* intragroup: Wilcoxon test, *p* intergroup: Mann–Whitney U test. The data are expressed as the median (interquartile range), Δ change between two measurements.

Abbreviations: NDI, Neck Disability Index; VAS, Visual Analogue Scale.

^∗^ *p* < 0.05.

CROM measurements are shown in Table 4. All CROM values increased significantly after the interventions in both groups (*p* < 0.05). There were no differences between groups in the improvement of CROM of lateral flexion, flexion, extension and right rotation. The CROM of left rotation increased significantly only in the kinesiotaping group (*p* = 0.001, Table 4).

**TABLE 4:** Comparison of CROM parameters over time.

CROM	Kinesiotaping group (*n* = 14) median (IQR)	Pilates group (*n* = 16) median (IQR)	*p* intergroup
Lateral flexion, right			
Baseline	37.30 (29.86–40.0)	37.65 (30.65–42.48)	
8 weeks	43.43 (36.85−53.75)	46.38 (41.94–48.54)	
Δ	8.5	8.23	0.755
*p* intragroup	0.005^∗^	0.001^∗^	
Lateral flexion, left			
Baseline	35.13 (28.68–40.44)	34.58 (32.95–42.73)	
8 weeks	46.05 (39.74–57.58)	44.73 (41.98–47.59)	
Δ	13.45	7.68	0.074
*p* intragroup	0.002^∗^	< 0.001^∗^	
Flexion			
Baseline	41.30 (39.50–46.13)	46.10 (40.83–49.06)	
8 weeks	60.88 (49.45–69.11)	58.05 (55.08–65.84)	
Δ	18.13	14.02	0.151
*p* intragroup	0.001^∗^	< 0.001^∗^	
Extension			
Baseline	47.73 (40.25–51.34)	52.55 (44.78–57.63)	
8 weeks	62.23 (58.41–72.80)	62.45 (59.65–71.96)	
Δ	17.90	11.95	0.383
*p* intragroup	0.001^∗^	< 0.001^∗^	
Rotation, right			
Baseline	62.60 (54.22–67.85)	69.30 (62.21–74.37)	
8 weeks	80.32 (71.90–83.56)	80.08 (74.53–84.20)	
Δ	17.28	9.02	0.074
*p* intragroup	0.002^∗^	< 0.001^∗^	
Rotation, left			
Baseline	61.40 (54.76–65.56)	72.57 (65.53−75.45)	
8 weeks	78.82 (71.67–83.41)	80.92 (74.09−82.5)	
Δ	19.17	8.13	0.014^∗^
*p* intragroup	0.001^∗^	< 0.001^∗^	

*Note:* *p* intragroup: Wilcoxon test, *p* intergroup: Mann–Whitney U test. The data are expressed as the median (interquartile range), Δ change between two measurements.

Abbreviation: CROM, cervical range of motion.

^∗^ *p* < 0.05.

Table 5 presents a comparison of neck muscle strength and core muscle endurance within and between the kinesiotaping and Pilates groups. Both intervention groups demonstrated statistically significant improvements in all outcome measures following the 8‐week intervention period (*p* < 0.05 for all intragroup comparisons). Notably, the greatest increases were observed in the prone bridge and sit‐up tests, indicating improvements in core endurance. Neck muscle strength parameters, including flexion, extension and lateral flexion, also showed significant enhancements in both groups. Despite significant improvements within each group, intergroup comparisons revealed no statistically significant differences in any parameter (*p* > 0.05).

**TABLE 5:** Comparison of neck muscle strength and core muscle endurance within and between the groups.

	Kinesiotaping group (*n* = 14) median (IQR)	Pilates group (*n* = 16) median (IQR)	*p* intergroup
Lateral flexion, right (kg)			
Baseline	6.73 (5.68–7.62)	6.20 (5.78–7.30)	
8 weeks	8.18 (6.70–10.07)	8.65 (7.30–10.31)	
Δ	1.08	2.30	0.212
*p* intragroup	0.002^∗^	0.001^∗^	
Lateral flexion, left (kg)			
Baseline	6.63 (5.33–7.50)	5.80 (5.20–7.08)	
8 weeks	8.18 (6.40–10.21)	8.25 (7.18–9.45)	
Δ	0.93	2.35	0.053
*p* intragroup	0.003^∗^	< 0.001^∗^	
Flexion (kg)			
Baseline	6.38 (5.15–7.76)	5.98 (5.32–6.73)	
8 weeks	7.00 (6.08–9.08)	7.5 (5.73–9.58)	
Δ	0.90	1.48	0.519
*p* intragroup	0.008^∗^	0.002^∗^	
Extension (kg)			
Baseline	7.65 (6.21–9.19)	7.10 (6.16–8.94)	
8 weeks	11.30 (8.23–12.54)	10.55 (8.60–13.00)	
Δ	1.19	1.76	0.394
*p* intragroup	0.001^∗^	< 0.001^∗^	
Prone bridge (s)			
Baseline	36.07 (30.69–53.24)	33.96 (27.45–37.34)	
8 weeks	55.56 (44.04–71.93)	53.28 (40.21–73.96)	
Δ	21.27	12.26	0.561
*p* intragroup	0.002^∗^	0.003^∗^	
Sits up, repetitions/30 s			
Baseline	10.50 (7.63–12.50)	9.25 (4.63–11.63)	
8 weeks	13.50 (10.88–16.63)	13.50 (9.75–16.00)	
Δ	4.25	4.75	0.369
*p* intragroup	0.001^∗^	< 0.001^∗^	

*Note: p* intragroup: Wilcoxon test, *p* intergroup: Mann–Whitney U test. The data are expressed as the median (interquartile range), Δ change between two measurements.

^∗^ *p* < 0.05.

## 4. Discussion

This randomised controlled trial aimed to compare the effects of Pilates exercises alone and Pilates combined with kinesiotaping application over 8 weeks in office workers with chronic neck pain. Our results showed significant improvements in neck pain, disability, CROM, muscle strength and core endurance parameters in both groups. Furthermore, intergroup comparisons revealed that kinesiotaping provided an additional benefit over Pilates only in terms of left neck rotation CROM. These findings indicate that Pilates exercises are an effective treatment method for managing chronic neck pain, but kinesiotaping may only play a complementary role in a specific plane of motion.

### 4.1. Pain and Disability

It is well known that prolonged sitting and poor posture among office workers can lead to cervicothoracic dysfunction [31]. Pilates exercises target the deep postural muscles to increase stability, which contributes to reducing pain perception [32]. The pain‐reducing effect of Pilates exercises can also be explained by the ‘gate control theory′. Pilates can suppress pain transmission at the dorsal horn level by increasing afferent signalling via proprioceptors [9, 33]. However, the reduction of psychological stress through breath control and mental awareness in Pilates may also indirectly raise the pain threshold [34, 35].

A number of studies in the literature suggest that Pilates exercises are an effective treatment for chronic neck pain. It has been found that Pilates reduces neck pain in office workers with chronic neck pain [36]. A 6‐week Pilates programme has been reported to reduce neck pain and disability in patients with chronic nonspecific neck pain [33]. Similarly, a study of patients with chronic neck pain found that Pilates and home exercise programmes reduced disability levels [37]. Byrnes et al. also reported positive effects on functional outcomes via increased motivation to exercise [38]. Our findings are consistent with these studies.

Previous studies have reported that, although kinesiotaping reduces pain in the short term, its effects on neck disability are limited [39, 40]. However, it has also been noted that applying kinesiotaping in addition to home exercises reduces pain and disability more effectively than exercise alone [41]. Contrastingly, Zengi et al. reported that kinesiotaping added to home exercise programmes was effective in managing neck pain in bus drivers; however, the addition of kinesiotaping was not more effective than exercise therapy alone, a finding that closely parallels our results [17].

Similarly, in our study adding kinesiotaping to Pilates did not provide any additional benefit in terms of neck pain or disability. This can be explained by the fact that Pilates has a very strong pain modulation effect, which masks the possible additional contribution of kinesiotaping.

### 4.2. CROM

It is well‐known that Pilates exercises improve proprioception by balancing the activity of agonist and antagonist muscles, thereby increasing the CROM. Şahiner et al. reported that Pilates exercises increased CROM in all directions except extension [33]. However, another study found that 12 weeks of Pilates training for chronic neck pain patients did not result in a change in CROM at the 6‐week follow‐up [34]. In our study, CROM was measured using a digital inclinometer, and a significant increase was observed in all directions. This result may be due to the exercises being applied regularly and under supervision, as well as the participants having similar postural risk profiles (i.e., similar occupational postural characteristics).

The literature on the effects of kinesiotaping on CROM is conflicting. Saavedra‐Hernández et al. found that applying kinesiotaping increased CROM in all directions except right and left rotation, in a manner similar to cervical manipulation [42]. However, Ay et al. reported that kinesiotaping only provided a limited contribution to the short‐term increase in CROM. They applied kinesiotaping to patients every 3 days for 15 days, reporting an increase in neck flexion and extension movements but no significant change in neck rotation and lateral flexion movements [39]. In our study, a significant increase in CROM was detected in both groups, but we found that kinesiotaping provided an additional benefit only for left rotation. This difference may be due to kinesiotaping increasing movement coordination through proprioceptive feedback and facilitating movement by reducing neck pain. While Pilates directly increases CROM by developing active muscle control and stability, kinesiotaping may facilitate movement through sensory stimulation.

### 4.3. Muscle Strength and Endurance

Pilates primarily increases the strength and endurance of the muscles around the torso by activating deep muscle groups, thereby improving motor control [43]. Ulug et al. compared the effects of Pilates, yoga and isometric exercises on patients with neck pain. They found that only the Pilates group showed an increase in the thickness of the semispinalis capitis muscle, as assessed by ultrasound [9]. In our study, significant increases in the strength of the neck flexor, extensor and lateral flexor muscles were obtained, as measured by a push–pull dynamometer. Additionally, core endurance values, as assessed by the prone bridge and sit‐up tests, increased similarly. Our findings are consistent with previous studies reporting increases in deep cervical flexor muscle endurance and core endurance [33, 44].

In our study, we observed a significant increase in muscle strength and endurance in the kinesiotaping group, which was similar to that seen in the Pilates group. Copurgensli et al. compared conventional treatment, conventional treatment supplemented with Mulligan mobilisation and kinesiotaping application in patients with cervical spondylosis, finding a significant increase in muscle strength in all groups. They also noted that the kinesiotaping group performed better than the other groups at increasing deep flexor muscle strength. The authors emphasised that kinesiotaping can modulate muscle activity through proprioceptive feedback, particularly enhancing the performance of deep flexor muscles [45]. Our results suggest that, when combined with Pilates′ effect of increasing muscle activation, this mechanism of kinesiotaping application may create a synergistic effect. To our knowledge, no studies have examined the direct effects of kinesiotaping on neck muscle endurance; therefore, our results are an important preliminary finding for future studies. Although muscle strength and endurance increased in both groups in our study, no significant difference was found between them. This suggests that Pilates is a powerful method for improving muscle performance independently. It is possible that the positive effects of kinesiotaping were overshadowed by the dominant effect of Pilates in this situation.

### 4.4. Occupational Rehabilitation Perspective

From an occupational rehabilitation perspective, chronic neck pain in office workers represents not only a clinical condition but also a major barrier to sustained work participation and occupational performance. The improvements observed in pain, disability, cervical mobility, muscular strength and endurance directly relate to essential work‐related demands such as prolonged sitting tolerance, postural control during computer‐based tasks and the ability to maintain functional performance throughout the workday. Pilates‐based interventions enhance postural stability, motor coordination and core endurance, thereby addressing the physical demands of sedentary occupations and facilitating the recovery of functional work capacity. Consequently, the present findings highlight the relevance of exercise‐based interventions within occupational rehabilitation programmes aimed at preventing work‐related disability and promoting long‐term work participation.

Collectively, the findings support the effectiveness of the structured Pilates programme, which was associated with consistent improvements in pain, disability, CROM, muscle performance and core endurance. Although kinesiotaping produced additional improvement in left cervical rotation, its overall contribution appeared limited when combined with an intensive exercise programme. This pattern indicates that active exercise‐based rehabilitation may play a more substantial role than passive adjunctive modalities in addressing the multidimensional impairments associated with chronic neck pain among office workers.

### 4.5. Clinical Significance

Chronic neck pain is one of the most common musculoskeletal problems experienced by modern office workers. However, the number of studies examining the effectiveness of Pilates exercises for office workers with neck pain is limited. Only one study has been found in the literature that evaluates the effect of Pilates exercises on neck pain in office workers, and this study focused solely on pain levels [36]. No study has examined its effects on neck disability, CROM, muscle strength and endurance in a multidimensional manner.

Our findings suggest that Pilates exercises can comprehensively improve pain, disability, muscle strength and endurance. This indicates that Pilates may be an effective approach to reducing work loss and improving quality of life. On the other hand, kinesiotaping may partially support treatment, particularly by increasing CROM. For these reasons, Pilates is recommended as a primary rehabilitation strategy for the clinical management of chronic neck pain, while kinesiotaping can be considered a complementary treatment method.

To our knowledge, this is the first randomised controlled trial to compare a supervised Pilates programme alone with Pilates combined with kinesiotaping in office workers with chronic neck pain. Previous studies have largely investigated these interventions separately and have primarily focused on pain and disability outcomes. In contrast, the present study evaluated a broader spectrum of clinically relevant parameters, including CROM, neck muscle strength and core endurance. Furthermore, by focussing specifically on office workers, a population exposed to prolonged sitting and sustained postural demands, the study provides evidence that is directly applicable to occupational rehabilitation settings. These findings contribute to the current literature by clarifying the additional value of kinesiotaping when combined with a structured Pilates programme.

### 4.6. Limitations

Our study has certain limitations. Firstly, the sample size was small, and as the follow‐up period was restricted to 8 weeks, the long‐term effects could not be evaluated. The fact that all participants were office workers employed at the same university also reduces the generalisability of the results. The relatively high dropout rate may have reduced statistical power and should be considered when interpreting the findings. Furthermore, while the evaluators and practitioners conducting the statistical analysis were blinded, the inability to fully achieve practitioner blinding in the Pilates and kinesiotaping applications represents a methodological limitation. Future studies should plan for larger samples, long‐term follow‐up and comparisons of different Pilates and kinesiotaping application protocols, as well as including different occupational groups.

## 5. Conclusions

The results of this study suggest that 8 weeks of Pilates exercises offer office workers with chronic neck pain a range of benefits and that kinesiotaping may only provide additional benefits for specific movements (particularly rotational ones). The comprehensive effects of Pilates on pain control, CROM, muscle strength and endurance highlight this method as a primary approach to chronic neck pain rehabilitation. From an occupational rehabilitation perspective, Pilates‐based exercises support work‐related functional capacity and may facilitate sustained work participation in sedentary occupations, with kinesiotaping serving as a complementary approach when required.

## Author Contributions

Abdulkadir Göz: writing–original draft, resources, methodology, investigation, formal analysis, conceptualisation, final approval.

Ali Mutlu: writing–review and editing, supervision, methodology, conceptualisation, final approval.

Evrim Göz: writing–project administration, review and editing, supervision, methodology, conceptualisation, final approval.

## Funding

This study was supported by Tarsus University Scientific Research Projects Coordination Unit (Grant Number: SBF.23.001).

## Ethics Statement

The study was approved by the Ethics Committee of Tarsus University on 25.05.2023 with decision number 2023/20. The Declaration of Helsinki was followed in the study.

## Consent

All participants signed informed consent.

## Conflicts of Interest

The authors declare no conflicts of interest.

## Supporting Information

Additional supporting information can be found online in the Supporting Information section.

## Supporting information


**Supporting Information** The supplementary file contains the Pilates exercises for Weeks 1–4 (Supporting Figures 1–3) and Weeks 5–8 (Supporting Figures 4–6).

## Data Availability

Data are available on request due to privacy/ethical restrictions.
