# Supplementary material for: Pilates and Kinesiotaping for Neck Pain, Disability and Cervical Function in Office Workers: A Randomised Controlled Trial
Source: Pain Res Manag. 2026 Jul 23;2026:1954204. doi: 10.1155/prm/1954204 (PMC13396692; doi:10.1155/prm/1954204)
Supplement: Supplementary file 1 — Supporting Information The supplementary file contains the Pilates exercises for Weeks 1–4 (Supporting Figures 1–3) and Weeks 5–8 (Supporting Figures 4–6). [file PRM-2026-1954204-s001.pdf]

PILATES AND KINESIOTAPING FOR NECK PAIN, DISABILITY AND CERVICAL FUNCTION IN OFFICE WORKERS: A RANDOMISED CONTROLLED TRIAL

Abdulkadir GÖZ, Ali MUTLU, Evrim GÖZ

PILATES TRAINING PROGRAM 1-4 WEEKS

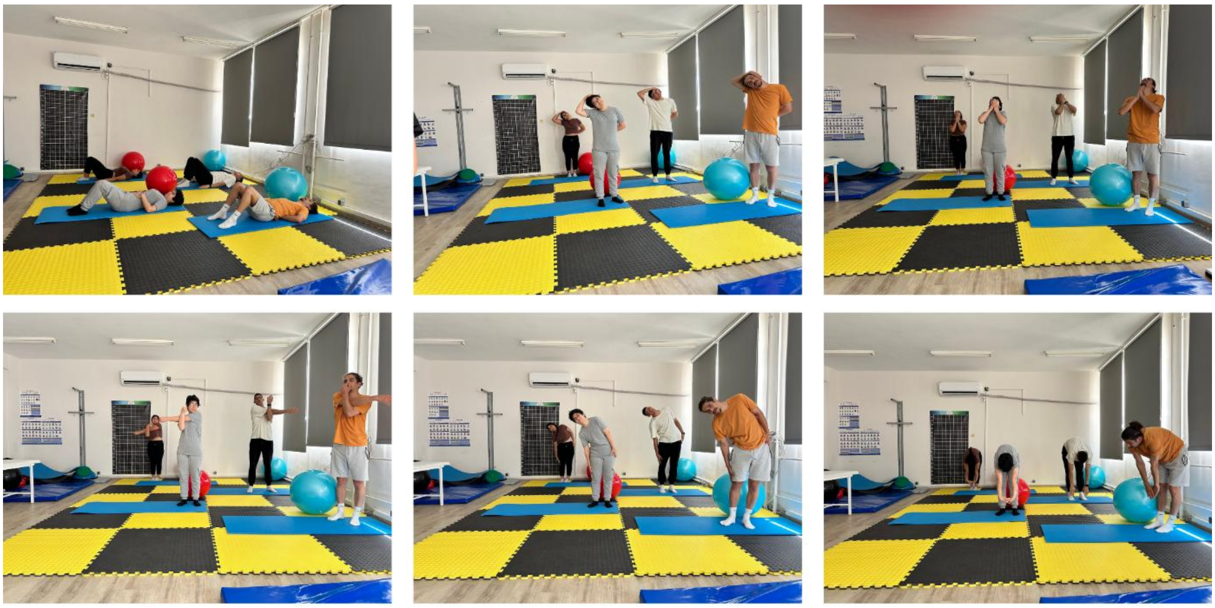

Supplementary Figure 1. Warm up exercises, 1-4 weeks

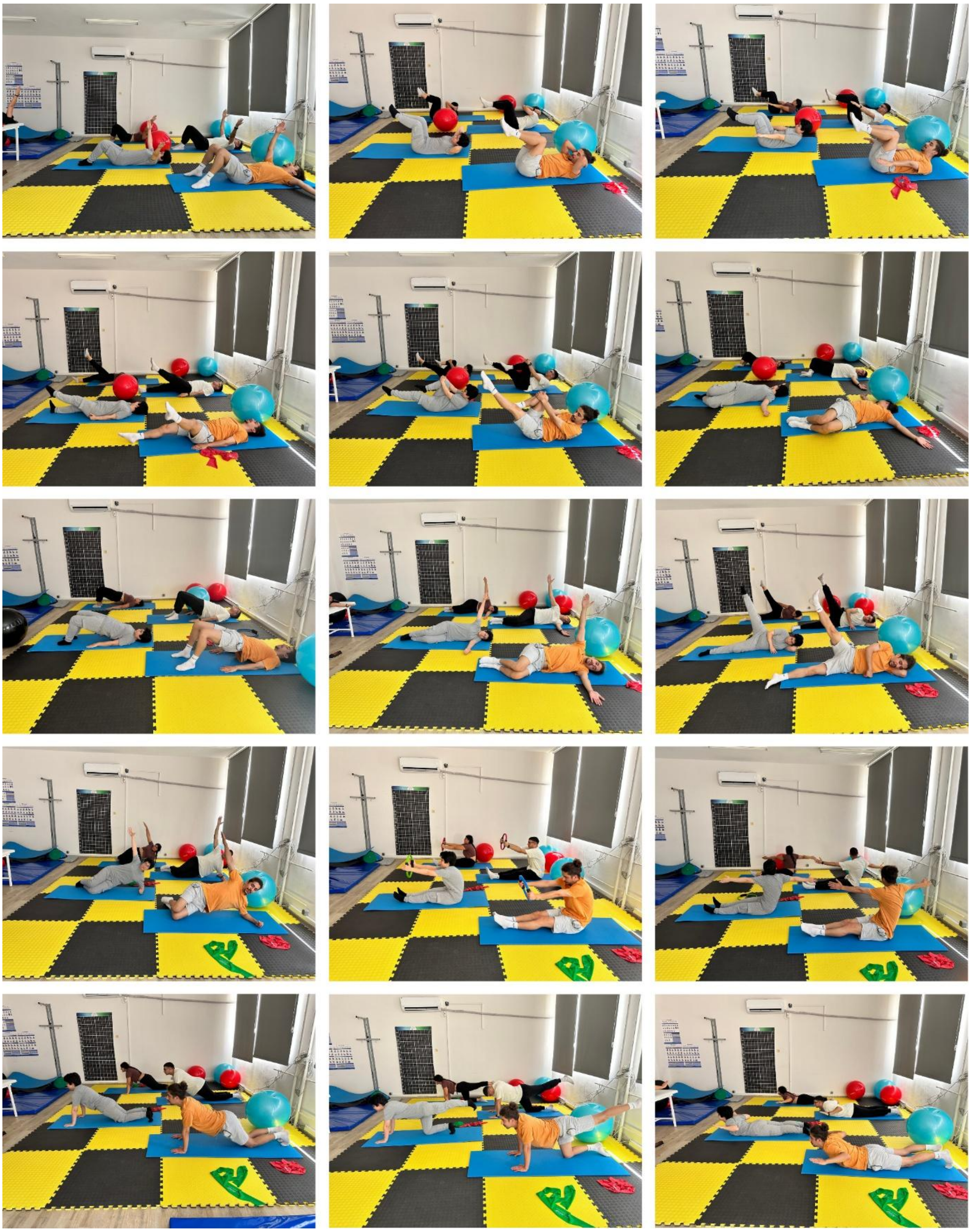

Supplementary Figure 2. Pilates exercises, 1-4 weeks

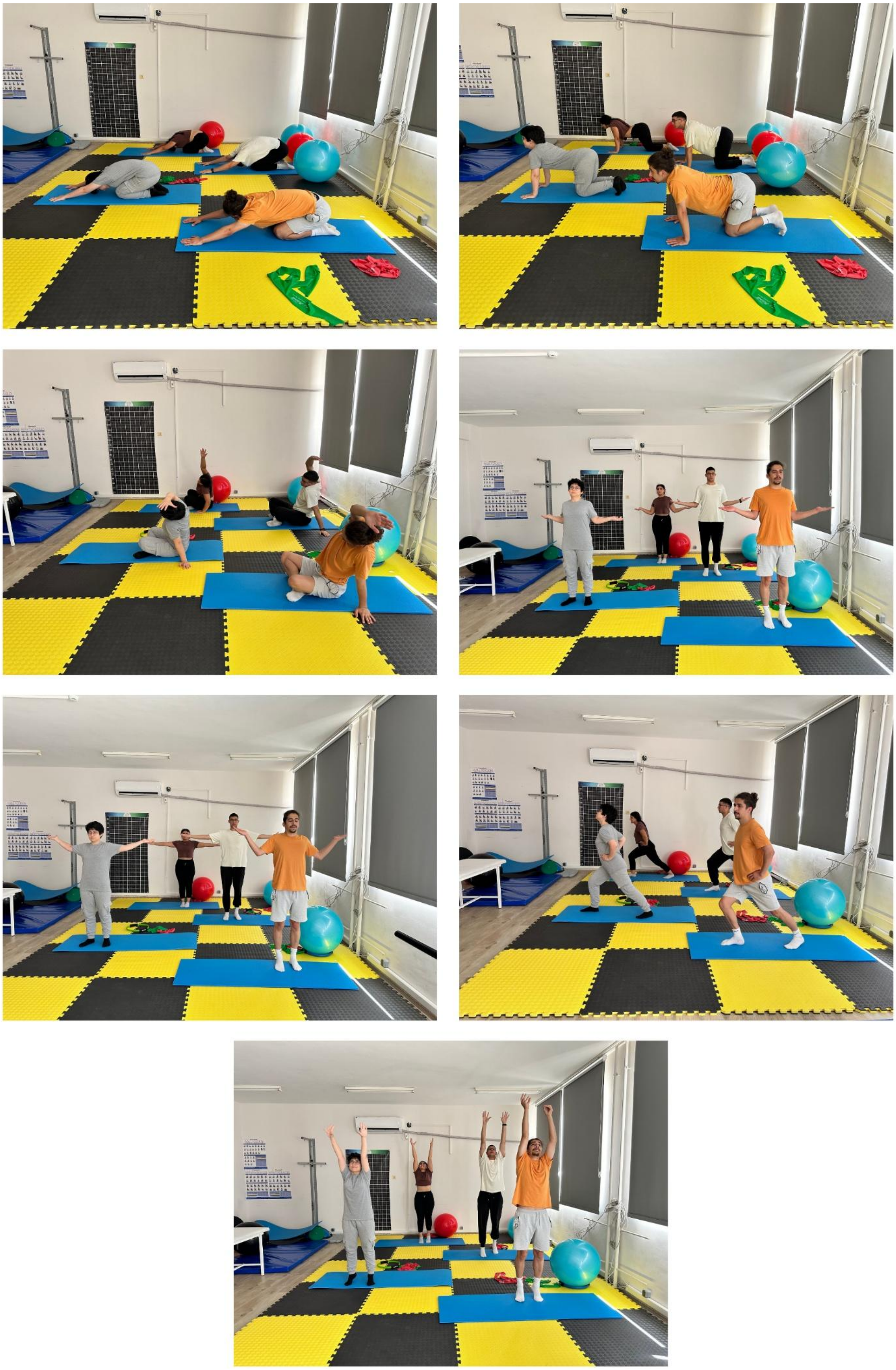

Supplementary Figure 3. Cool-down exercises, 1-4 weeks

PILATES TRAINING PROGRAM 5-8 WEEKS

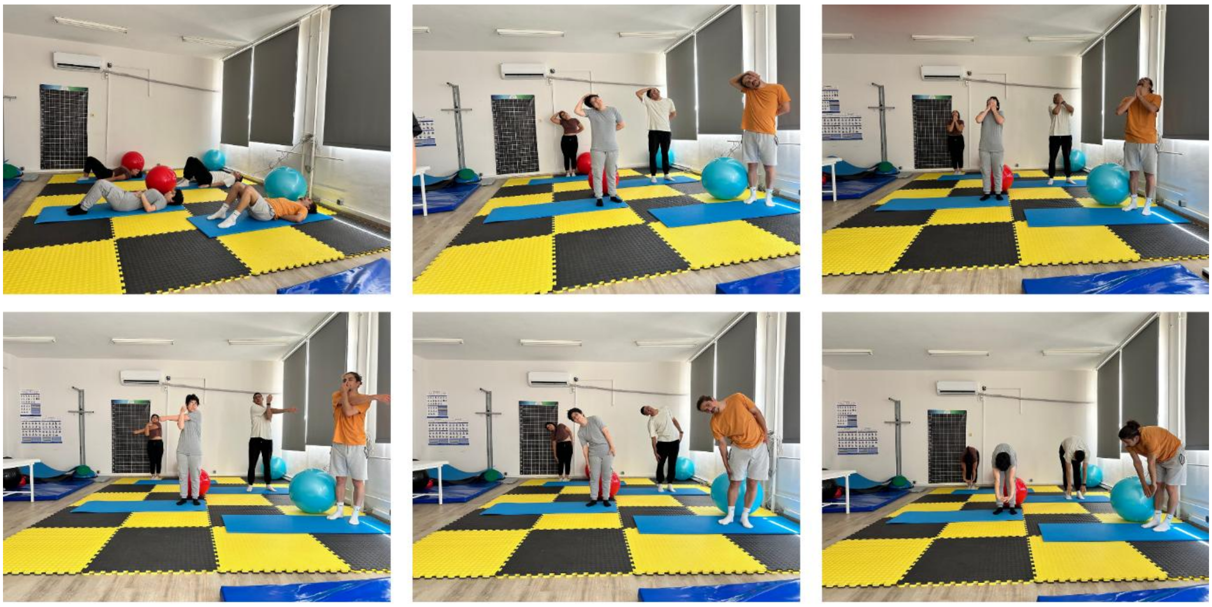

Supplementary Figure 4. Warm up exercises, 5-8 weeks

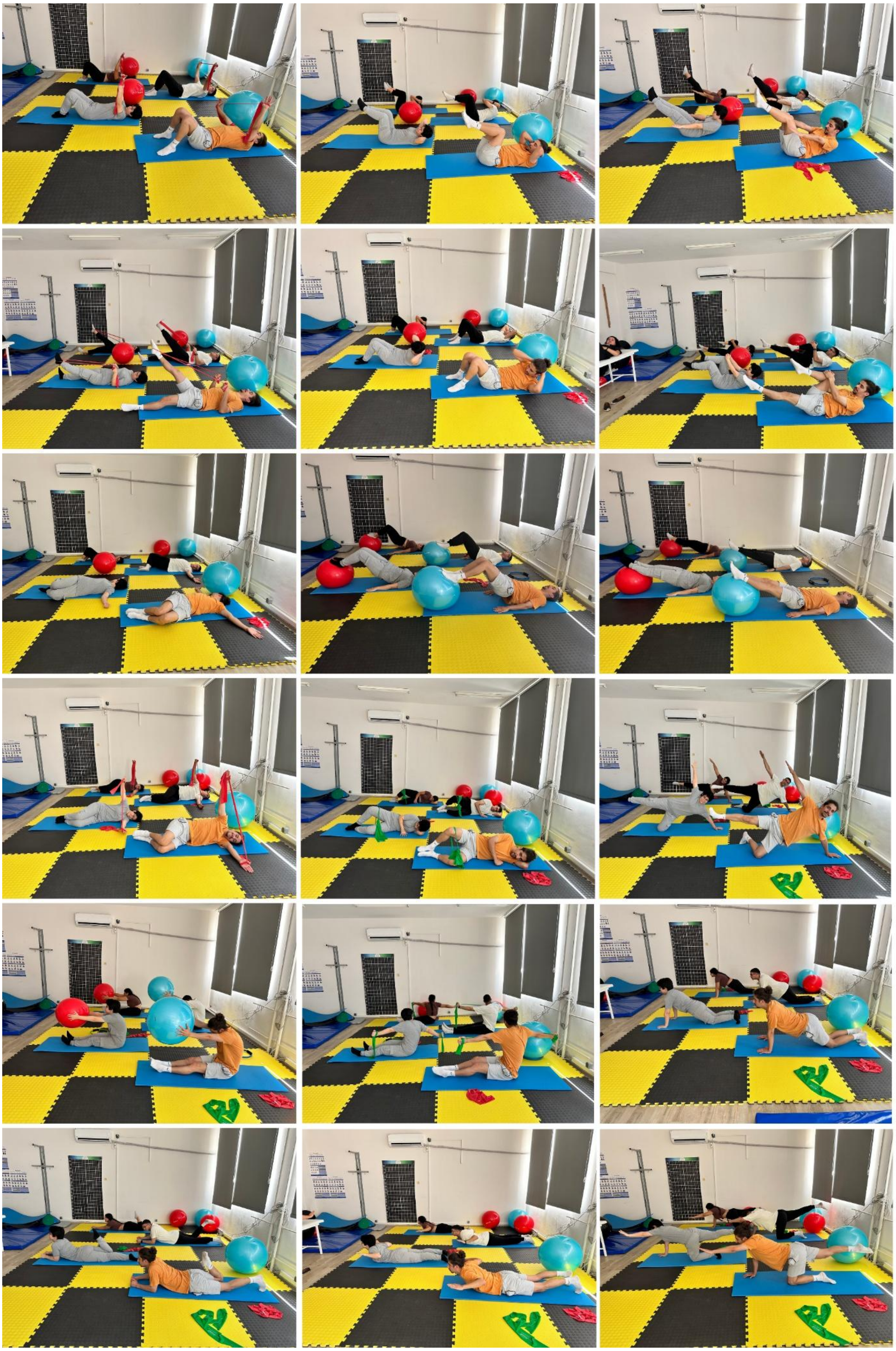

Supplementary Figure 5. Pilates exercises 5-8 weeks

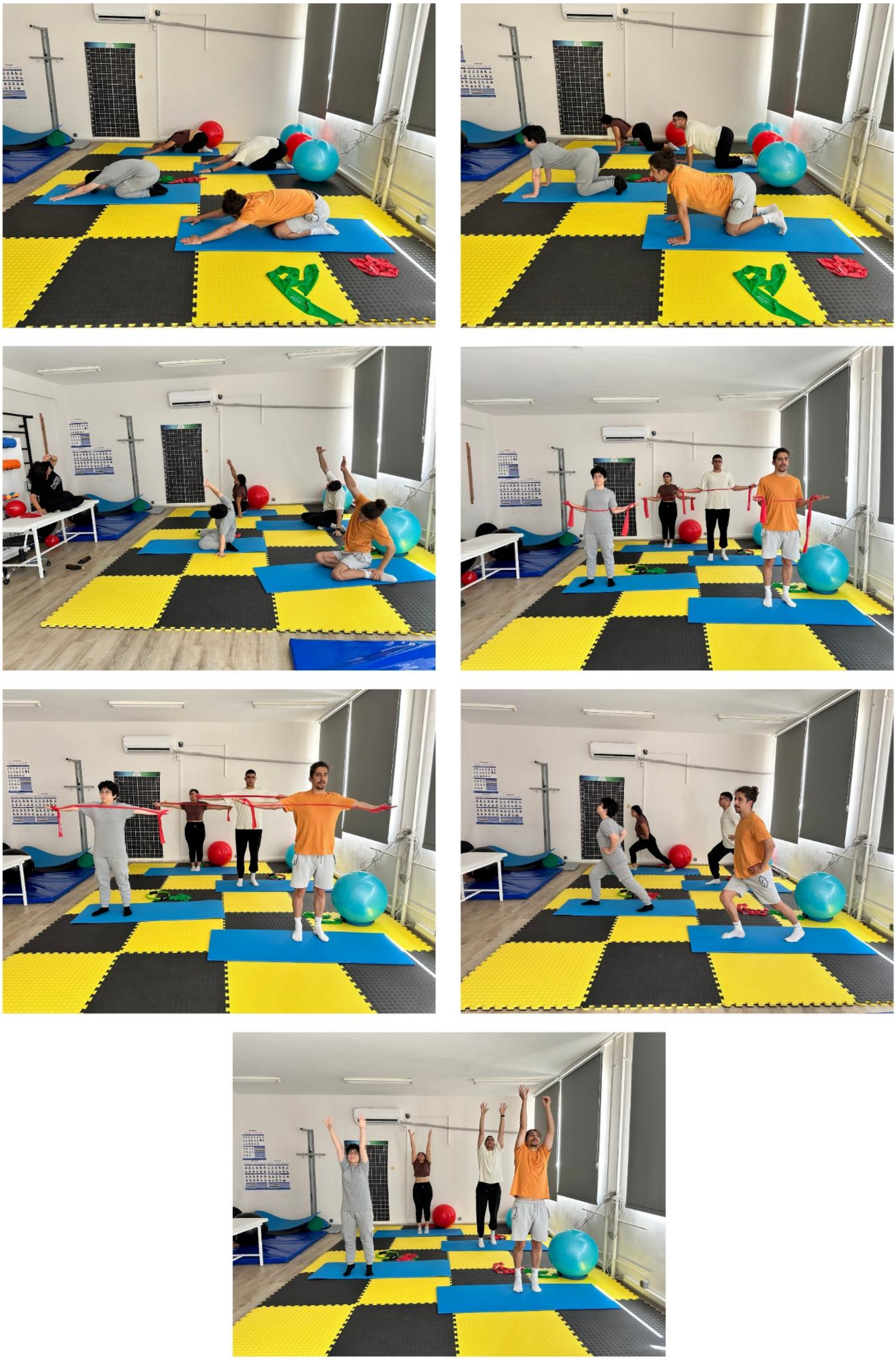

Supplementary Figure 6. Cool-down exercises, 5-8 weeks
